# A service evaluation of the clinical contingencies implemented during a Linac replacement programme

**DOI:** 10.1093/bjro/tzaf006

**Published:** 2025-04-11

**Authors:** Chloe Wells, Mike Kirby

**Affiliations:** Radiotherapy Department, The Christie NHS Foundation Trust, Manchester M20 4BX, United Kingdom; School of Allied Health Professions and Nursing, Institute of Population Health, University of Liverpool, Liverpool L69 3GB, United Kingdom

**Keywords:** Linac replacement programme, clinical contingencies, service evaluation

## Abstract

**Objective:**

A Linac Replacement Programme (LRP) was completed to ensure continuity of treatment whilst maintaining the highest standards of care. Clinical contingencies were devised to mitigate the impact of unscheduled interruptions during the LRP. This service evaluation was undertaken to appraise the effectiveness of contingencies on treatment delivery (TD) during the LRP.

**Method:**

The oncology management system MOSAIQ was used to generate reports of treatment adjustments. These reports were then generated for Linac service history in the 2019-2020 year for comparative analysis and causative factors in Linac breakdowns. Adjustments to treatment were analysed for each patient.

**Results:**

Of the 855 patients receiving treatment during the LRP, 184 were impacted in some way. Of these, 113 experienced some increase in overall treatment time (OTT); 742 (86.8%), therefore, experienced no increase in OTT, through deployment of clinical contingencies or not encountering machine breakdown during their treatment schedules. Throughout the LRP, Conebeam CT (CBCT) faults were the primary cause for machine closure. Due to this, breast patients remained on treatment at a higher rate than prostate patients who required 3D-geometric verification prior to TD.

**Conclusions:**

This project highlighted the importance of preparation for CBCT faults and patient categorization in the development of contingencies. The extended dose and fractionation 60 Gy in 20# presented increased opportunities for cancellation in prostate patients, however, the use of MV imaging to assess patient set-up enabled continuation of TD. Increases in OTT could not be eliminated completely, however, for 21.5% of patients who experienced treatment adjustments the implementation of contingencies effectively prevented them exceeding Royal College of Radiologists guidance of 2-day extension in OTT.

**Advances in knowledge:**

We believe this radiographer-led project is the first service evaluation reporting the practical effects on treatment of a LRP and impact of clinical contingencies used to mitigate and limit unscheduled interruptions in treatment and minimize the extension of OTT for patients during the transition.

## Introduction

Radiotherapy (RT) is a rapidly evolving field in oncology treatment, with ceaseless progression and technological development increasing the efficacy of treatment. Due to the continual advancement of techniques inevitably Linear Accelerators (Linacs) will require improvement and replacement to ensure best patient care is maintained.^1^ The developments of more advanced techniques results in increased conformity in plans and reduction in toxicity to surrounding healthy tissues. The Royal College of Radiologists (RCR)^2^ highlighted their concern in their published priority document, stating that the rapidity with which RT technology is advancing, particularly within the last decade, many RT centres within the United Kingdom are beginning to become outdated and becoming unable to deliver “optimal patient care.”

Time is a crucial element in radiation oncology.^3–5^ As highlighted by Fowler and Lindstrom in 1992,^6^ there is evidence to show that the prolongation of treatment through missed fractions can compromise local control and overall achievement of therapeutic outcomes.^5^^,^^6^ Unscheduled breaks in treatment allow increased time for repopulation of cancer cells, therefore, it was essential that patients only miss treatments where there was no alternative, in line with the RCR guidance.^7^^,^^5^

Matched machines are Linacs considered dosimetrically identical and allow the transfer of patients between machines without the need to alter treatment plans, thus reducing impact on patient treatment and improving the efficiency of the department.^8^ When Linac breakdowns (LBs) occur, as outlined by the RCR,^7^ the ideal resolution is transfer to a matched machine. In a 2-Linac centre, when only 1 machine is delivering treatment, this is not possible. Missed treatments may have to be replaced by additional treatments in out-of-service hours, adding additional fractions to the end of the treatment or bidaily treatments.^7^ Each of which could be affected by staff availability, funding, service capacity, and patient compliance.

The Christie network presently comprises 15 Linacs across a main site^9^ and three 2-Linac Satellites. Linac replacement was considered before any satellites were opened, as part of the operational procedures for the network (eg, regarding costs, staff rotation and resources, ability to transfer Category 1 patients and other contingency plans, etc).

In anticipation of Linac replacement at the Christie at Oldham (the first Satellite established), options were considered to transfer and/or install new Linacs across the network; 1 option was close the Satellite completely, whilst new Linacs were installed. However, consecutive replacement was chosen. Though longer in timescale, this would minimize patient disruption whilst maintaining services closer to patients, reducing travel and maintaining patient compliance with treatment schedules. Continuity of other holistic and supportive services, such as onsite Macmillan support and specialist Physiotherapy (especially for breast patients), would also be preserved. In 2023, the Linac Replacement Programme (LRP) was completed with the installation of 2 new Elekta Versa HD Linacs. Clinical contingencies were devised to mitigate the impact of any unscheduled interruptions during the LRP.

Prolongation of treatment of any length can compromise therapeutic outcomes for Category 1 patients as defined by the RCR guidance^7^ therefore, treatment delivery (TD) for these patients was relocated to alternative Christie radiotherapy departments. This project aimed to appraise the degree to which clinical contingencies devised by the multidisciplinary team decreased the impact on TD. To answer this, 2 primary objectives were evaluated. Firstly, how effectively the contingency protocols put in place minimized unscheduled breaks. Further, how the LRP data compared with cancellations in a year when both machines were fully functioning (2019-2020).

Although each department is unique in terms of configurations, resources, capacity, workloads, etc, this service evaluation should benefit the community by expanding on published experiences of linac replacement, with a particular applicability for those RT networks with Satellite Centres or other networked provision.

## Methods

**Figure 1.**
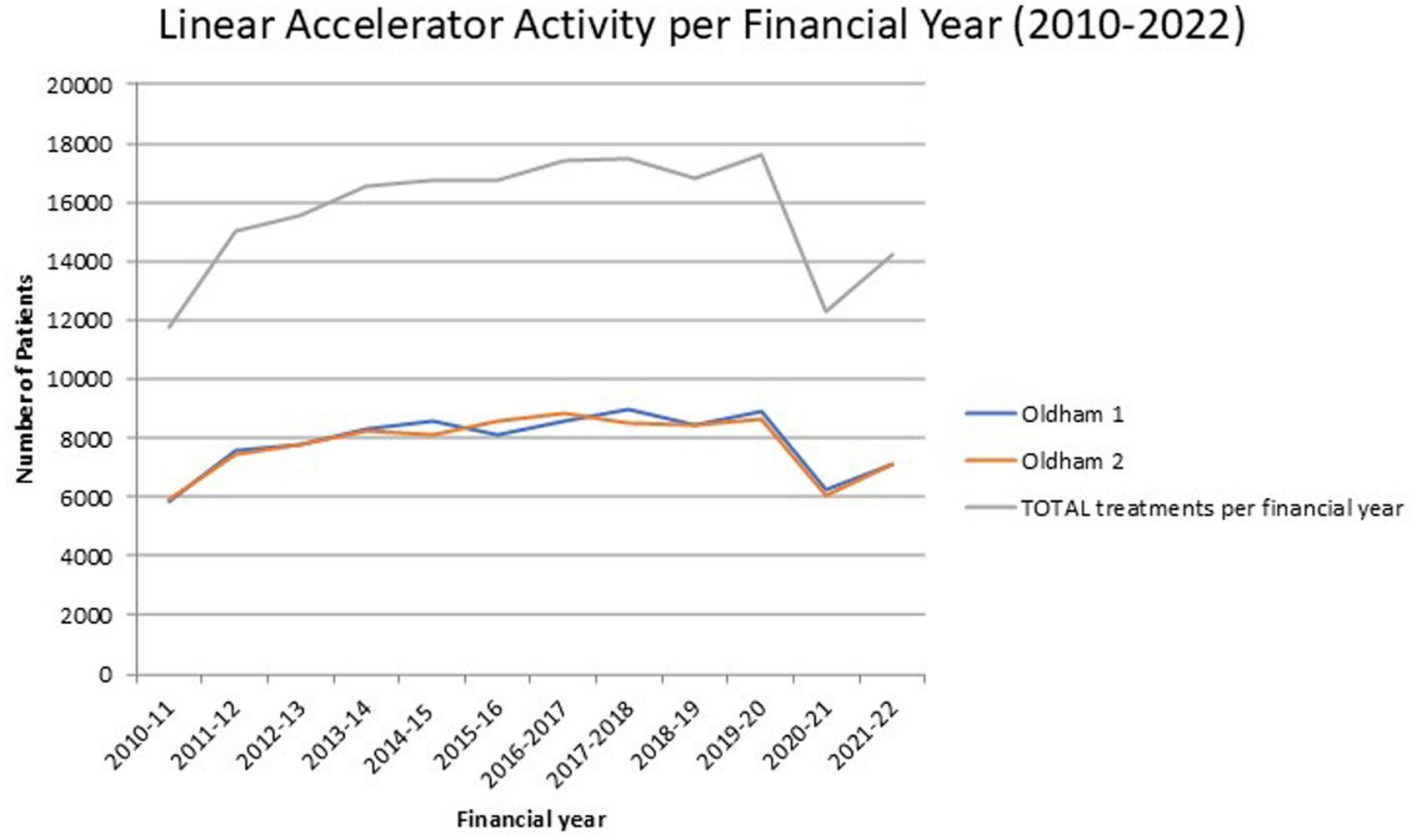
Oldham Linac performance per FY from 2010 to 2022, showing the drop in Linac activity due to the COVID-19 pandemic. Oldham 1 and 2 are the respective names for the 2 Linacs housed in the RT department at Oldham.

During the LRP, 855 patients received RT. The oncology management system MOSAIQ was used to generate reports to identify all incidents of treatment adjustment (Table 1). The same detailed reports were generated for Linac service history in the 2019-2020 financial year (FY) for comparative analysis. In the 2019-2020 FY, 132 patients were either delayed or cancelled for Linac services. This financial period was chosen as the best comparative measure, since the following year, the COVID-19 pandemic significantly impacted patient treatment numbers (Figure 1). During the pandemic patients were diverted to active surveillance which, along with the implementation of the FAST-Forward trials findings,^9^ saw a consequential decrease in departmental footfall to reduce infection control risks. The data from the pandemic years would not have been comparative due to the reduced level of Linac activity in that period (Figure 1).

**Table 1.** Outline of the reports run to identify all instances of machine breakdown and patient cancellations.

Report	Selection criteria	Filters applied
PATIENTS TREATED REPORT	Treatment Record for April 28, 2022 New Starts - April 28, 2022-April 21, 2023	New starts at other Clinical Sites, for example Withington Macclesfield Salford
Patient Cancellation Report	HCAN LB - April 28, 2022-April 21, 2023 DNT Technical - April 28, 2022-April 21, 2023 DNT—imaging issue - April 28, 2022-April 21, 2023 HCAN Linac Service - April 28, 2022-April 21, 2023 HCAN Mosaiq/Service - April 28, 2022-April 21, 2023 HCAN imaging issue - April 28, 2022-April 21, 2023	Patient unable to tolerate treatment position Patient required Repeat RT Planning Scan Patient did not attend appointment.
Machine Breakdown Report (2022-2023)	Machine Breakdown Treatment Machine Not Available Machine Service	Non-Applicable
Machine Service Report (2019-2020)	Machine Service	Non-Applicable

Inclusion criteria for patient cancellation reports was clinically cancelled appointments; Did Not Treat (DNT) and Healthcare Cancellation (HCAN) these appointments were altered on clinical rationale rather and personal patient basis.

During the LRP, the clinical contingencies devised and implemented included the treatment of Category 1 patients in other sites of the Christie network; the use of orthogonal 2D MV imaging in the event of significant Conebeam CT (CBCT) breakdown. Where necessary, 2-fractions per day (a minimum of 6 hours apart) were considered, depending upon capacity and resources.

The LRP population was then subdivided into 2 groups; those whose TD was not impacted, and those for whom treatment was adjusted. Causative factors in LBs and adjustments to treatment were analysed for each patient. Patients who had multiple adjustments were identified to ascertain changes in overall treatment time (OTT).

For LBs in particular, data was sought both from the Mosaiq records, but also directly from fault logs and in consultation with Linac Engineers and Physicists; to identify major Linac and Imaging faults as well as other instances (such as hardware/software upgrades) which would impact patient treatments.

Approvals for this service evaluation were sought from and granted by the Clinical Lead at The Christie at Oldham, the Education Team at The Christie NHS Foundation Trust and the trust’s Quality Improvement and Clinical Audit Team. All report data from Mosaiq was extracted fully anonymized.

## Results

**Figure 2.**
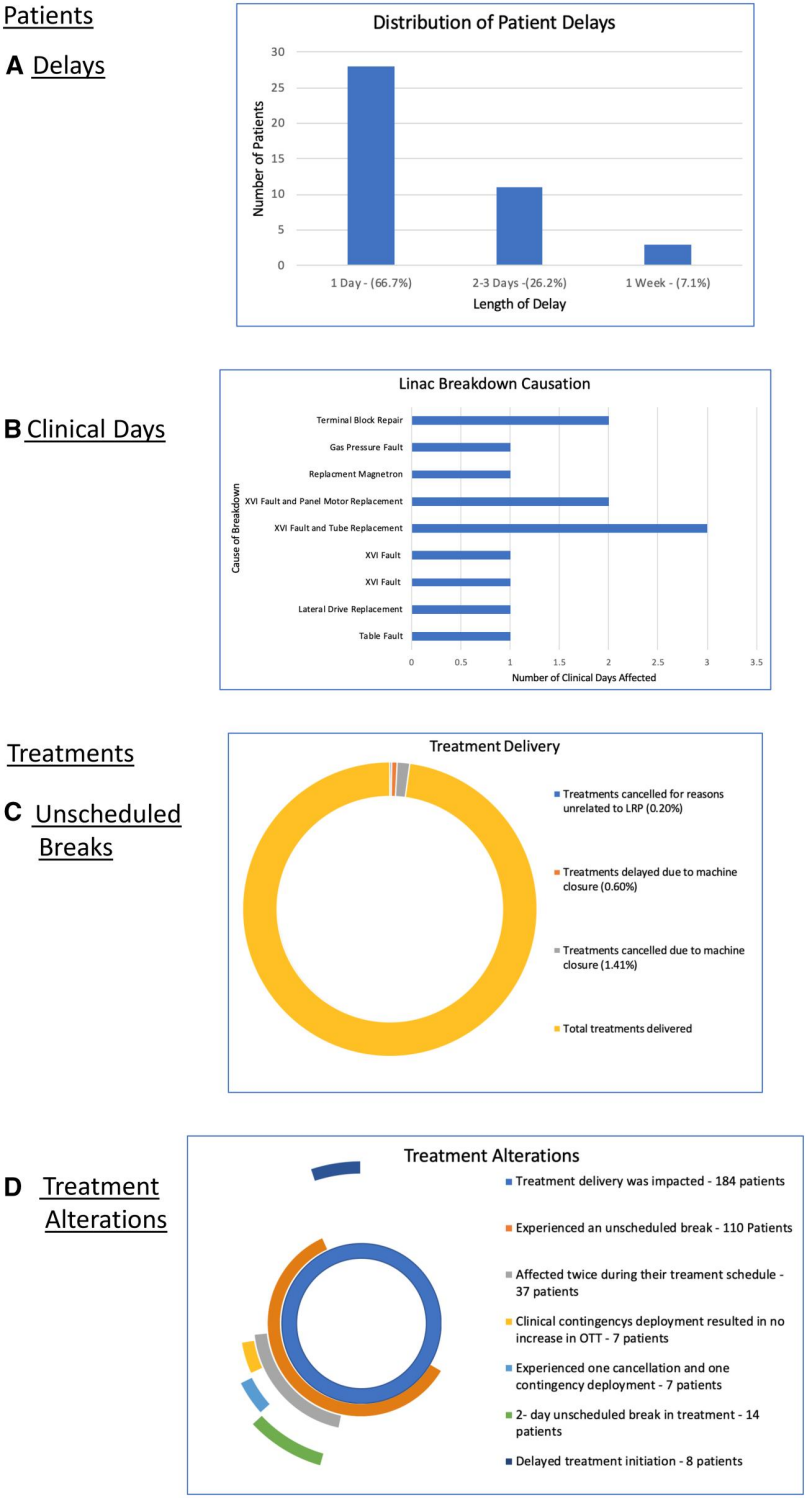
The data presents details for the consequences of LBs during the LRP, with several patients falling into multiple categories. (A) Graphic representation of the distribution of patient delays. (B) Causation of LBs and clinical days impacted. (C) Overall TD, and the proportions impacted during the LRP. (D) Graphic representation of Treatment alterations, detailing number of patients, and alteration incurred.

Of the 855 patients who received treatment during the LRP, 184 were impacted in some way. Of these, 113 experienced some increase in OTT; 742 (86.8%), therefore, experienced no increase in OTT, through either not being impacted by a machine fault/breakdown, or deployment of a contingency during a fault. Patient delays experienced during the LRP primarily were 1-day delays to initiation of treatment (Figure 2A). Twenty-nine patients were treated with an alteration in imaging protocol which resulted in no increase in OTT. Thirty-seven patients were affected twice during their treatment schedule; 7 experienced no increase in OTT due to deployment of the imaging contingency; 7 had treatment cancelled resulting in a 1-day increase in OTT and 14 experienced a 2-day unscheduled break in treatment.

Distribution of causes of LB and impact on service delivery regarding clinical days affected is displayed in Figure 2B with XVI breakdowns and terminal block repair causing prolonged clinical disruption. Figure 2B shows the main faults causing significant delays and downtime (1 day and above) to the machine and patient treatments. Some of the XVI faults meant the machine was still useable (with the imaging contingency of MV instead of kV imaging), but these are not easily filtered out from the Mosaiq reports. Through the duration of the LRP, TD continued unaffected for 97.8% of treatments (fractions), with only ∼2% of treatments altered due to LB (Figure 2C). The Treatment alteration breakdown in terms of patients is displayed in Figure 2D, showing, for example, those having delayed treatment initiation, those with a 2-day unscheduled break, etc

**Figure 3.**
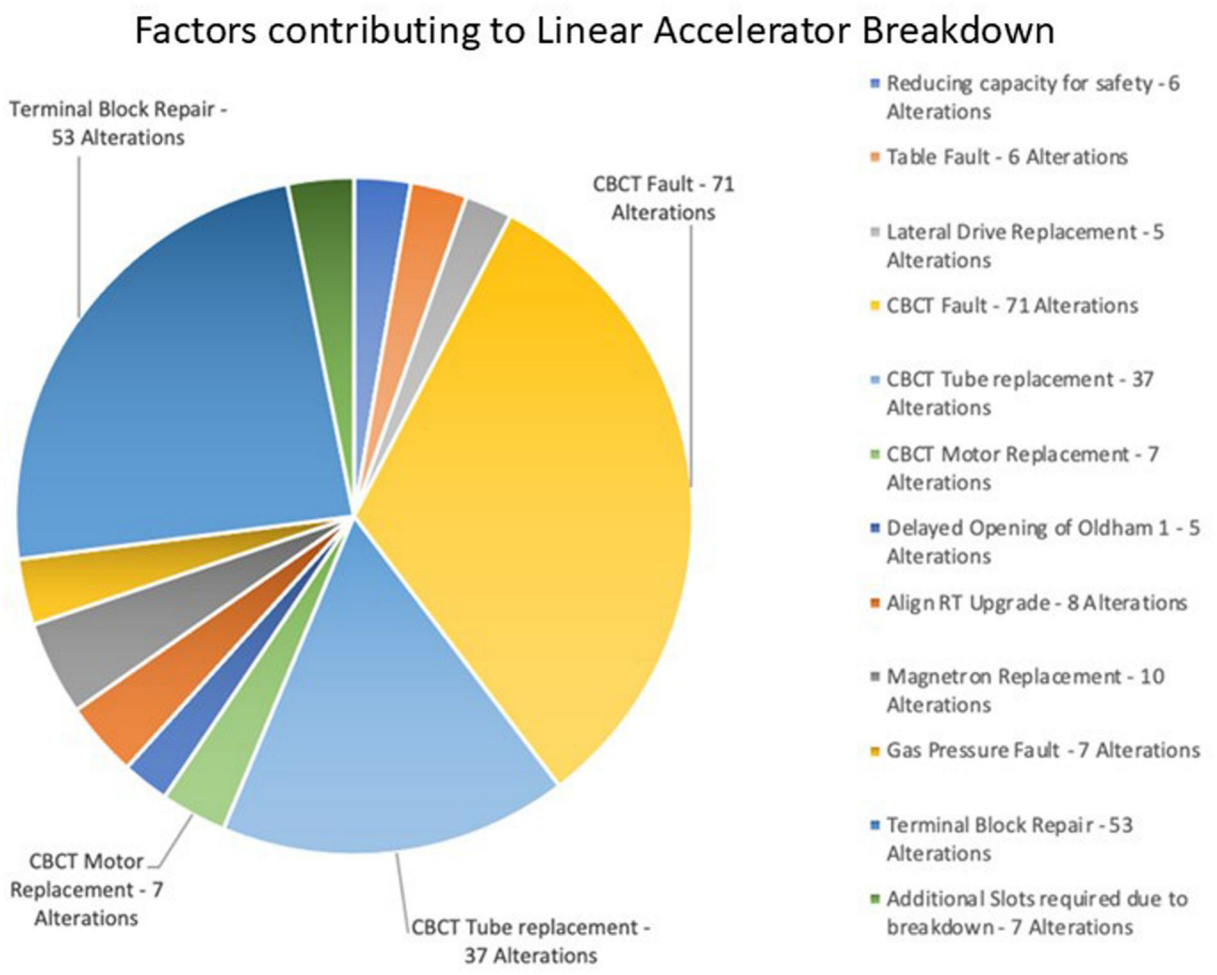
Factors contributing to Linear Accelerator Breakdowns and adjustment to TD during the LRP. The majority (115/222) (52%) were related to CBCT volumetric on-treatment imaging equipment.

Throughout the LRP, LBs were a leading factor of treatment adjustment, as shown in Figure 3, which shows expanded detail on the machine faults and other significant occurrences causing patient alterations. CBCT faults were the primary cause for machine closure, resulting in 115/222 (52%) of treatment alterations. Due to the prevalence of CBCT faults, breast patients remained on treatment at a higher rate than prostate patients who required 3D CBCT verification. Some instances (such as the AlignRT upgrade) were not machine faults *per se*, but still had a service impact.

**Figure 4.**
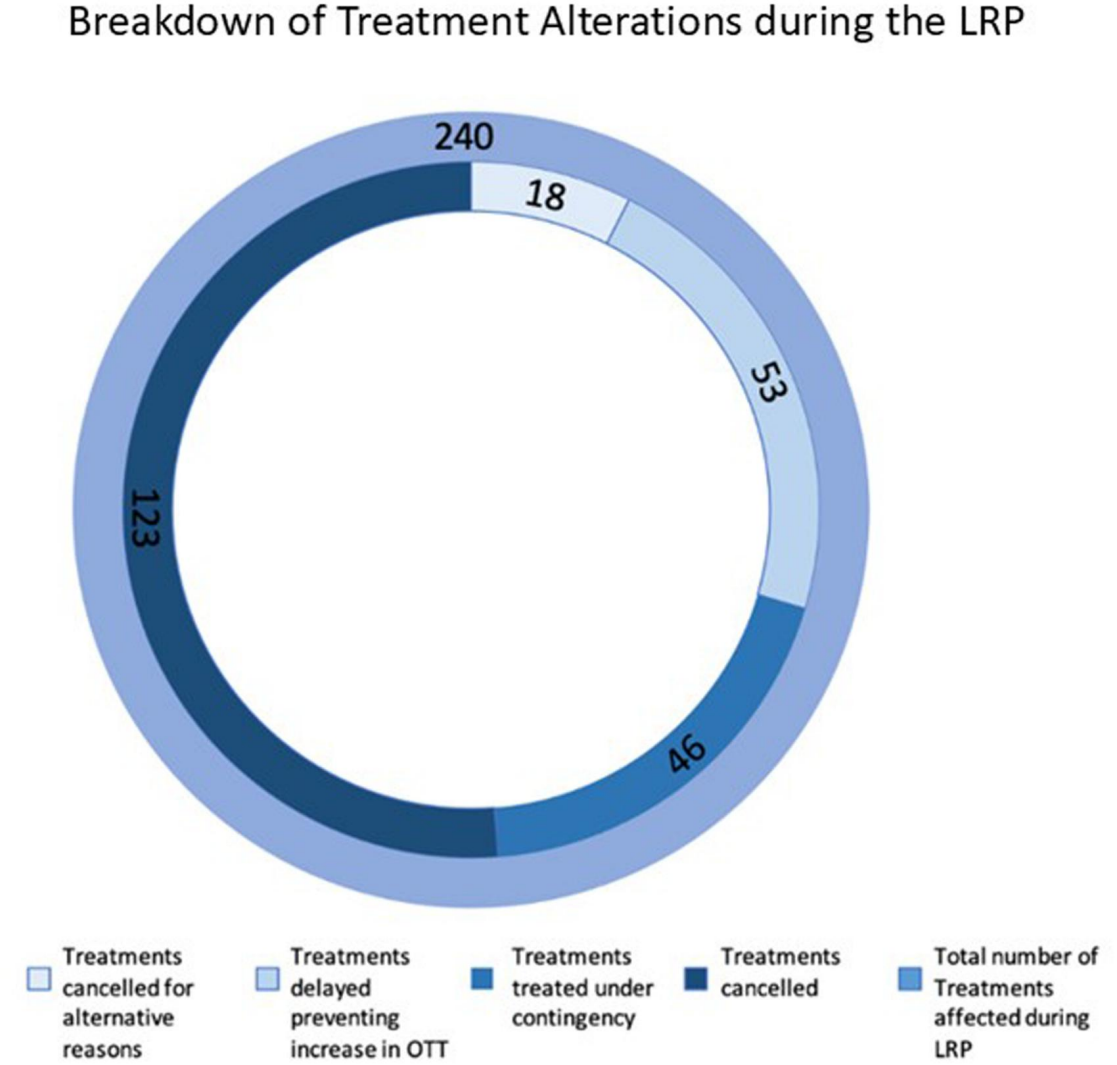
Graphic representation of the number of treatment alterations during the LRP. The outer ring represents the total number of treatment alterations, while the inner ring demonstrates the breakdown of which alteration was employed for each treatment.

Over 8700 treatments (fractions) were delivered during the LRP. Of these 240 treatments (2.75%) were altered due to LBs (Figure 4). Of these, 18 (0.2%) were cancelled for reasons unrelated to the LRP (Figures 2C and 4); 99 (1.1%) were either delayed or treated under contingency to prevent an increase in OTT; 123 (1.4%) were cancelled due to machine closure (Figure 4). Twice daily fractionation was not used as no patients exceeded a 2-day break.

**Figure 5.**
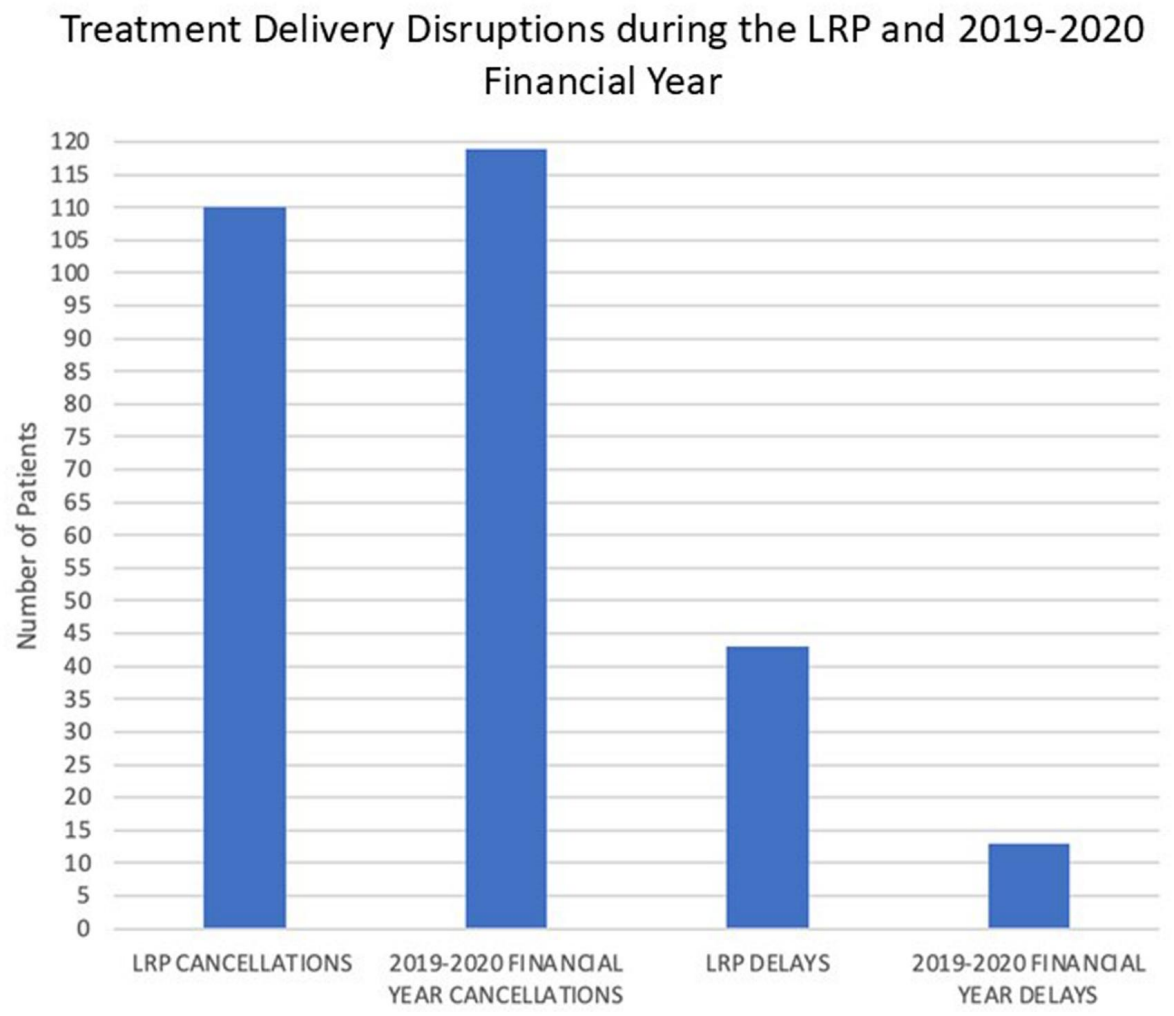
Comparison of the number of patients cancelled and delayed before the start of treatment during the LRP with the data from the 2019 to 2020 data.

The comparative analysis of the LRP data with the 2019-2020 data, demonstrated that the deployment of contingencies prevented the number of patients experiencing cancellations exceeding the number recorded during a fully functional FY (Figure 5). However, there was a predominance of prostate patients in the LRP data, with 58.7% of affected patients receiving treatment for prostate cancer in comparison with 39.3% in 2019-2020 data. The single Category 1^10^ patient who experienced an unscheduled break evidences the importance of the relocation of service delivery for these patients.

## Discussion

### Linac breakdown impact on TD

Breakdowns that required machine closure for urgent repair impacted patients across all diagnoses. In these incidents it was not possible to implement clinical contingencies except delaying new patients. During the LRP, 86.8% of all patients who received treatment completed their treatment schedule with no increase in OTT. Of the patients impacted by the machine fault or breakdown, (45.7%) completed treatment within 1 day of target in line with RCR guidance. All unscheduled breaks were compensated to ensure patients received their entire prescription.^7^ In the results, 18.9% of patient’s increase in OTT was limited to 1 day due to the implementation of the imaging clinical contingency (Figure 2D). While there is evidence to suggest the timing of the unscheduled break within the treatment schedule is a prognostic factor in the achievement of therapeutic outcomes^11^ more recent RCR guidance disagrees,^7^ therefore, this data were not collected in this study. The patients who experienced alterations to treatments unrelated to the LRP, such as anatomical changes requiring re-scan, were not included to remove potential misrepresentation of the impact of LBs during the LRP. Between delays and imaging contingencies 36.9% of patients experienced no increase in OTT during the LRP, further evidencing the significant role clinical contingencies played in reducing unscheduled breaks.

### Patient disease groups

The primary group of patients impacted during the LRP was prostate patients, due to the use of CBCT for 3D image verification in TD. Seventy-nine Prostate patients experienced either a delay or cancellation during their treatment schedule, in comparison with 55 breast patients. Standard dose and fractionation of prostate treatment is 60 Gy in 20#; for breast cancers recent application of the FAST-Forward 26 Gy in 5# has reduced the number of patients receiving the standard 40 Gy in 15#.^9^ There is currently no published data on impact of unscheduled breaks for breast treatments with these prescriptions.^7^ The relatively recent implementation of the FAST-Forward trials findings^9^ has meant there are no findings regarding implications of unscheduled breaks in such short dose and fractionations for this subgroup of patients, with the only literary evidence regarding breaks in breast irradiation concerned with 5-week prescriptions, which are no longer standard of care.^5^^,^^12^

For Prostate patients the research on the impact of unscheduled breaks is ambiguous, primarily focused on longer treatment interruption and techniques that were prevalent prior to the introduction of 60 Gy in 20#, their findings are limited in applicability to the dose and fractionation prescribed to patients receiving treatment at this RT centre.^13^ Despite this, with the lowest dose per fraction and longest timescale for TD, an unscheduled break will be less detrimental for a prostate patient than for a 5# breast. The dose and fractionation of 26 Gy in 5# has a secondary benefit, due to reduced fractionation there is less opportunity for a breakdown to occur during their TD. This is evidenced through 14.4% (55) of the 381 breast patients treated during the LRP, experiencing a delay or cancellation during their treatment schedule, compared with 34.3% (79) of the 230 prostate patients. This protective characteristic of the prescription for breast patients may have contributed to the disparity between prostate and breast patients impacted by LBs in addition to the reliance of prostate treatments on CBCT image verification.

### CBCT linac breakdowns

Image guided radiotherapy (IGRT) remains a fundamental aspect of the safe and accurate delivery of RT.^14^ The use of IGRT minimizes the probability of a geometrical miss. When IGRT capabilities are lost, geometrical miss and uncertainty of toxicity to organs at risk (OARs) are a primary concern. The CBCT disruption in this LRP presented a challenge to staff. Whilst the deployment of clinical contingency increased the workload for the radiographers on set, it reduced the number of possible cancellations by 20.7%. Without the implementation of this contingency an additional 29 patients (15.7%) would have experienced an unscheduled break in their treatment schedule. In the event of a CBCT fault, breast patients and patients who are not routinely imaged daily with CBCT were fast-tracked through to treatment enabling workflow to continue and reduce the impact of delays experienced within the department.

Two-dimensional planar imaging when compared with 3D standard for daily IGRT has reduced visualization meaning that whilst it may reduce the probability of a geometrical miss, it does not eliminate it completely. Gregoire et al.,^15^ highlighted the clinical dose required to successfully irradiate cancer cells is beyond the tolerance of healthy cells. Subsequently incorrect irradiation could result in acute toxicity of surrounding OARs with severe consequences for patients. The use of the orthogonal 2D MV imaging allowed visualization of the bony anatomy in the sagittal and coronal planes to be translated into couch move corrections.^16^^,^^17^ This contingency provided essential anatomical information for high-precision RT without detriment to surrounding OARs. During the LRP, no margin changes were undertaken in the planning process in the event of 2D MV imaging deployment. The increased preparatory workload was considered compared with the likely times the contingency would be needed. The reduction of cancellations of 20.7% demonstrates the clinical contingencies employed during the LRP reduced unscheduled breaks. 32 of the 110 prostate patients impacted during the LRP had the imaging contingency deployed during CBCT faults, reducing unscheduled breaks by almost a third. Although 32 prostate patients were impacted, only 46 (0.5%) of 8700 treatments (fractions) delivered in total during the LRP were treated under contingency (Figure 4). The contingencies deployed during CBCT faults were not always applicable when breakdowns resulted in machine closure. Breakdowns affected 13 clinical days (Figure 2B), with TD continuing due to contingency deployment on 39% of these. This demonstrates the efficacy of the imaging contingencies deployed.

The delay of initiation of treatment for patients prevents an increase in OTT. The research into the impact of delay in the initiation of RT primarily focusses on the extended hospitalization following surgery, extended healing process and the impact of delays in Category 1 patients. For the Category 2 and 3 patients that received treatment during the LRP the impact of delays of 1-7 days are not well-elucidated. Wang et al.^18^ examined the delay between mastectomy and postmastectomy RT. While this study focused on sociodemographic and postsurgical complication factors rather than machine breakdown, the findings are applicable to breast patients receiving treatment during the LRP. While there is evidence that a delay in RT is not related to an increased risk of local recurrence,^19^^,^^20^ the importance of the timely delivery of RT highlighted by the RCR^6^ is reinforced in Wang et al.’s research.^18^

### Comparison with 2019-2020 data

The number of patients affected during the LRP was comparable with the 2019-2020 FY (Figure 5). A comparison of patient disease groups showed that there was a larger disparity between prostate and breast patients cancelled during the LRP than during the 2019-2020 FY, where they were virtually equal: 52 prostate and 54 breast patients. This comparison is only concerned with patients cancelled for Linac services, as a result there may have been patients cancelled due to LB during the 2019-2020 FY that have not been captured in the data. However, this would have been limited during this year with 2 functioning Linacs, with patients able to transfer to the matched machine in line with RCR guidance.^7^

The ratio of treatment alterations was different. During the 2019-2020 FY with 2 functioning Linacs 17 578 treatments were delivered, with 8700 delivered during the LRP. The number of patients cancelled during the LRP where only 1 Linac was available was commensurate with the 2019-2020 data. However, the number of treatment alterations was greater during the LRP, due to the inability to transfer to a matched machine in the event of a breakdown and the increased incidence of patients experiencing more than 1 cancellation though treatment. Forward planning of maintenance schedules during 2019-2020 prevented multiple treatment alterations for patients.

During the LRP there were 42 patients who experienced delays to the start of their treatment in comparison with 13 in 2019-2020. While preferential to an increase in OTT, delays must be minimized to ensure treatment planning scans accurately represent the disease during TD and to prevent compromise of disease control particularly with radical treatments.^21^ The LRP delays (Figure 2A) were primarily of 1 day (66.7%) with a maximum delay of 1 week (7.1%) the latter having planning scans subsequently adjusted to deliver an accurate disease representation. Hunter^21^ examined the detrimental impact delays in initiation of treatment can have on disease control. While useful for informing clinical decision making and supportive of RCR guidance on unscheduled delays, this article primarily focused on Category 1 cancers, and is, therefore, limited in its applicability to the delays of the Category 2 and 3 patients who received treatment during this LRP. The clinical contingency of diverting Category 1 patients resulted in no Category 1 patients having the start of treatment delayed. Assessment of patient categorization demonstrated that for Category 1 patients the diversion to alternative TD sites protected them from unscheduled breaks. Linac downtime was the primary causative factor in treatment cancellations and prevention of the implementation of clinical contingencies.

### Limitations

This project aimed to assess the effectivity of the clinical contingencies implemented during the LRP. It also aimed to review them using previous literature in this area. However, the lack of comparative literature was a primary barrier to establishment of a robust evidence base. The RCR guidance provided a platform for the creation of clinical contingencies, however, in the assessment of current literature the lack of peer reviewed articles presented a challenge when verifying this study’s findings. While the findings of this project will provide a base for future replacement programmes by documenting the clinical implications experienced by patients during an LRP where only 1 Linac was available, further research into this area would allow comparison and confirmation of results.

The data collection, analysis, and interpretation of results was undertaken by a single radiographer, raising the possibility of human error and bias occurring in these processes. The lack of previous literature and data in this area mitigated any bias that could have been generated through examination of earlier statistics and conclusions.

As mentioned earlier, our experiences here are within a large network delivery model across (now) 4 sites and, therefore, the results presented will be applicable first to other networks with Satellite Centres or networked provision. Most aspects of economic costs for hardware, software, licences, staff resources, etc are included in business planning for on-going replacement of equipment. Initial planning always considered the ability to treat Category 1 patients at main site, and that now includes all sites, where appropriate: with a rotation of staff around the network to help with changes in clinical and scientific workloads during the LRP.

## Conclusions

This service evaluation of the clinical contingencies implemented during the LRP was necessary to ensure quality patient care and inform future decision making. This project highlighted the importance of preparation for CBCT faults and patient categorization in the development of future clinical contingencies. It must be accepted that treatment cancellations cannot be eliminated entirely within a 2-Linac centre. Therefore, the priority was minimizing the impact of unscheduled breaks. The use of MV imaging to produce assessment of patient set-up enabled continuation of TD. Imaging contingencies effectively minimized the number of patients who experienced a 2-day break. For the 21.5% of patients (184) for whom treatment adjustment was employed, the clinical contingencies effectively prevented them exceeding the RCR guidance of 2-day extension in OTT. An LRP is necessary in the lifespan of Linacs. Individual hospitals have undertaken LRPs, developing clinical contingencies within national guidelines and department protocols. However, there remains little documented evidence on contingency planning or impact of the LRP on TD. While further examination is required to validate the initial findings, and other departments must consider their own unique configurations and resources, this evaluation provides valuable evidence for future standards for clinical contingencies in LRPs.

Our service evaluation has demonstrated that, whilst unscheduled breaks cannot be eliminated during an LRP, due to the unpredictable nature of LBs, the impact can be minimized using clinical contingencies. For future replacement programmes, it may be advantageous to examine the faults frequently reported on the Linacs due for replacement. This process may, alongside the findings of this study, aid fault forecasting, enabling clinical contingencies to be refined and scheduling improved further to limit the effect of faults and breakdowns on the patient disease groups.
